# EXPERIENCES OF LESBIAN, GAY, BISEXUAL, TRANSGENDER, AND/OR QUEER (LGBTQ) OLDER ADULTS WITH SERVICE PROVIDERS

**DOI:** 10.1093/geroni/igac059.164

**Published:** 2022-12-20

**Authors:** Rajean Moone, Barbara Gordon, Rajean Moone

## Abstract

Many lesbian, gay, bisexual, transgender, and/or queer (LGBTQ) older adults have experienced a lifetime of oppression and discrimination that often results in significant health, economic, and social disparities in comparison to their peers. Historically labeled as illegal, immoral, and/or sick, LGBTQ older adults often fear, hide, and distrust health care and other social services providers. This symposium will highlight contemporary research on the experiences and perceptions of LGBTQ older adults in relation to service providers. The first presentation will contextual factors that influence the healthcare utilization of LGBTQ middle-aged and older adults. The second presentation will explore older adult couples’ experiences of minority stress with service providers and effects on their relationships. The third presentation will explore explore the beliefs, experiences, and needs related to housing and housing discrimination in LGBTQ older adults living in coastal North Carolina. The fourth presentation will explore panel data of older LGBTQ adults residing in the U.S South and examine the effects of access to an LGBTQ affirming provider on health service utilization and aging outcomes.. The final presentation will explore the association between increased awareness, understanding, and belief in the Undetectable equals Unstransmittable campaign and having an LGBTQ affirming care provider. The symposium will conclude with a discussion on policy and practice impacts for aging services and health care providers.

